# Genomic safety, bacteriocinogenic potential, and probiotic-associated traits of *Enterococcus lactis* LBM-M3D isolated from the swine gastrointestinal tract

**DOI:** 10.3389/fmicb.2026.1920982

**Published:** 2026-09-03

**Authors:** Fernando Moises Mamani Sanca, Carlos Emilio Cabrera Matajira, Sebastián Bermúdez-Puga, Taís Mayumi Kuniyoshi, Mauro de Medeiros Oliveira, Iago Rodrigues Blanco, Marcos Camargo Knirsch, José Manuel Domínguez, Andrea Micke Moreno, Alessandro de Mello Varani, Ricardo Pinheiro de Souza Oliveira

**Affiliations:** 1 Department of Biochemical and Pharmaceutical Technology, Faculty of Pharmaceutical Sciences, São Paulo University, São Paulo, Brazil; 2 Department of Agricultural and Environmental Biotechnology, College of Agricultural and Veterinary Sciences, São Paulo State University (UNESP), São Paulo, Brazil; 3 Department of Chemical Engineering, Faculty of Sciences, Vigo University, Campus of Ourense, Ourense, Spain; 4 Department of Preventive Veterinary Medicine and Animal Health, School of Veterinary Medicine and Animal Sciences, University of São Paulo, São Paulo, Brazil

**Keywords:** bacteriocin, *Enterococcus lactis*, lactic acid bacteria, probiotic bacteria, swine

## Abstract

Swine production is vital for global food security; however, its sustainability is threatened by the overuse of antibiotics, which contributes to the emergence and dissemination of multidrug-resistant pathogens. Probiotics and bacteriocin-producing bacteria are promising strategies to support animal health and reduce antibiotic dependence. In this study, *Enterococcus lactis* LBM-M3D, isolated from the porcine gastrointestinal tract, was evaluated for *in vitro* antimicrobial activity, tolerance to simulated gastrointestinal conditions, genomic safety features, and bacteriocinogenic potential. The strain inhibited several Gram-positive swine-associated pathogens, including *Streptococcus suis*, *Staphylococcus hyicus*, *Listeria monocytogenes*, and *Enterococcus faecalis*. In addition, LBM-M3D remained viable under simulated gastrointestinal stress conditions, including exposure to pH 3.0 and 0.5% bile salts. Whole-genome sequencing identified four bacteriocin-like sequences, corresponding to enterolysin A, mundticin ATO6, enterocin SE-K4, and enterocin P. No acquired antimicrobial resistance genes were detected in plasmid-associated regions, whereas resistance- and virulence-associated determinants were exclusively chromosomally encoded. Although *E. lactis* is not currently included in the EFSA Qualified Presumption of Safety (QPS) list due to limited species-level safety data, our genomic analyses provide strain-level evidence supporting the safety of LBM-M3D. Overall, these findings indicate that LBM-M3D possesses probiotic-associated characteristics and represents a promising candidate for further probiotic development in swine production, pending additional functional validation, with potential applications in sustainable livestock systems.

## 1 Introduction

Animal production is fundamental to global food security, as it provides essential proteins and supports agricultural economies worldwide. Ensuring the sustainability of livestock systems, particularly in tropical regions, requires integrated strategies aligned with the One Health approach, which recognizes the interconnection between animal, human, and environmental health (Sacarrão-Birrento et al., 2024). As the global population is projected to exceed 9 billion by 2050, the need to increase food production further intensifies sustainability and public health challenges (Cole et al., 2018). In this context, the development of natural and sustainable alternatives to improve productivity and disease control in animal production systems has become increasingly urgent.

Within this scenario, swine production represents one of the most economically and strategically important livestock sectors worldwide. Swine farming accounts for more than one-third of global meat production, serving as a cornerstone of food security, agricultural economies, and international trade (VanderWaal and Deen, 2018). However, infectious diseases remain a major constraint on swine production. The globalization of the industry has facilitated the emergence and spread of numerous pathogens, posing significant challenges to animal health and food security (VanderWaal and Deen, 2018). One of the most widely adopted strategies for disease prevention and growth promotion has been the use of antibiotics, which have been employed since the 1930s. Nevertheless, their extensive use in animal production has become a major driver of the emergence and dissemination of multidrug-resistant bacteria (Ferri et al., 2017). In Brazil, the world’s fourth-largest pork exporter, with production reaching 5.3 million tons in 2024 [Associação Brasileira de Proteína Animal (ABPA), 2026], several studies have documented significant changes in antimicrobial resistance profiles. *Staphylococcus hyicus* has shown increased resistance to fluoroquinolones, lincosamides, and pleuromutilins, while resistance to florfenicol increased from 1.3% in the 1980s to 74.7% in 2012 (Moreno et al., 2022). Similarly, *Streptococcus suis*, a bacterium naturally present in the swine microbiota and capable of causing systemic infections, has exhibited increasing resistance to multiple antibiotics. One study reported that 72.1% of *S. suis* isolates were multidrug resistant (Matajira et al., 2019). Finally, *E. faecalis*, a common inhabitant of the swine gastrointestinal tract, was reported for the first time in 2017 to harbor linezolid-resistant strains isolated from healthy pigs carrying the cfr gene, which encodes a ribosomal methyltransferase that confers resistance to phenicols, lincosamides, pleuromutilins, streptogramin A, and oxazolidinones (Filsner et al., 2017). Therefore, the continued reliance on conventional antibiotics is becoming increasingly unsustainable.

As a consequence of this scenario, there has been growing interest in alternative strategies capable of maintaining animal health and productivity without contributing to the development of antimicrobial resistance. Natural resources, including plant extracts, organic acids, and essential oils, are increasingly being explored as alternatives to reduce antibiotic use and improve swine health (Li et al., 2021; Nhara et al., 2024). Among these approaches, probiotics have attracted particular attention because of their ability to modulate the intestinal microbiota, enhance immune responses, and improve overall animal well-being.

Among the microorganisms with probiotic potential, lactic acid bacteria (LAB) have received considerable attention owing to their beneficial effects on intestinal health and animal performance. However, before a strain can be considered a potential probiotic, its safety must first be thoroughly demonstrated. The application of probiotics requires rigorous safety assessment, as recommended by regulatory agencies such as the European Food Safety Authority (EFSA). The EFSA’s Qualified Presumption of Safety (QPS) status is a key tool for evaluating the safety of microbial species used in food and feed [EFSA Panel on Biological Hazards (BIOHAZ) et al., 2025]. Because probiotic safety is strain-specific, and because some species lack QPS status or sufficient species-level safety information, comprehensive strain-level safety characterization is essential for any microorganism intended for probiotic use. LAB, particularly species of *Lactobacillus*, *Limosilactobacillus*, *Enterococcus faecium*, *Pediococcus*, and *Bifidobacterium*, have been extensively investigated for their probiotic potential in swine (Myhill et al., 2021; Kaewchomphunuch et al., 2022; He et al., 2023; Zimmermann et al., 2024). Within this group of LAB, the genus *Enterococcus* has attracted increasing interest because of its dual relevance in both food biotechnology and public health. Although the genus *Enterococcus* plays an important role in food biopreservation, some species also include strains associated with human infections and the dissemination of antimicrobial resistance. The high genetic similarity between *Enterococcus lactis* and *E. faecium*, particularly in analyses based on the 16S rRNA gene, hindered the taxonomic distinction between these species for many years (Botina and Sukhodolets, 2006). Subsequently, *E. lactis* was formally described as a distinct species based on isolates obtained from raw milk cheeses (Morandi et al., 2012). More recently, comparative genomic studies demonstrated that strains previously classified as belonging to *E. faecium* Clade B actually correspond to *E. lactis*, which exhibits a lower prevalence of hospital-associated markers, acquired antimicrobial resistance genes, and virulence factors than the major clinical clades of *E. faecium* (Belloso Daza et al., 2021; Ross et al., 2025).

Collectively, these findings suggest that *E. lactis* may represent a promising candidate for the development of novel probiotics. However, the safety and functional characterization of *E. lactis* remain insufficiently explored at the strain level. These findings underscore the importance of further characterizing the probiotic potential of *E. lactis*, thereby supporting its evaluation for future biotechnological applications. Against this background, and considering the urgent need for sustainable alternatives to antibiotics in swine production, the present study characterizes the *E. lactis* LBM-M3D strain isolated from swine (*Sus scrofa*) to evaluate its safety and probiotic potential.

## 2 Materials and methods

### 2.1 Animal selection and sampling procedures

All procedures involving animals were approved by the Committee for Ethics in the Use of Animals (CEUA) under protocol number 7738030225. Cecal content samples were collected from three clinically healthy adult swine (*S. scrofa*), approximately 30 months of age. None of the animals had received antimicrobial treatment prior to sample collection. All animals belonged to commercial breeds (Landrace × Large White) and were part of a semi-intensive confinement system at the Swine Culture Facility of the USP “Fernando Costa” Campus in Pirassununga, Brazil. The animals were fed a diet consisting of ground corn (76%), soybean meal (22%), and a vitamin-mineral premix (2%). Samples were collected aseptically and transported to the laboratory under refrigeration (4 °C).

### 2.2 Bacterial isolation, strains, and culture conditions

The isolation of lactic acid bacteria (LAB) followed the methodology described by Schirru et al. (2012), with some modifications. Briefly, serial decimal dilutions (10^−1^ to 10^−7^) were prepared in saline solution (0.85% NaCl) at a 1:10 ratio, and 100 μL aliquots from each dilution were spread onto MRS agar (de Man, Rogosa and Sharpe) (BD Difco™, Strasbourg, France) supplemented with cycloheximide (0.1 g/L) to inhibit fungal growth. Plates were incubated at 37 °C for 24 h under two atmospheric conditions: (i) ambient aerobic conditions and (ii) in a sealed jar containing a lit candle to generate microaerophilic conditions (approximately 15–17% O₂ and 3–5% CO₂). This parallel incubation strategy was adopted to maximize the recovery of cultivable bacteria with different oxygen requirements. Plates showing the highest colony diversity were selected, and five morphologically distinct colonies from each plate were subjected to phenotypic characterization. The recovered LAB isolates were subsequently screened for antimicrobial activity against the selected indicator pathogens and evaluated for hemolytic activity on blood agar as an initial safety criterion. Priority was given to isolates that exhibited inhibitory activity against more than one swine pathogen of interest while showing a non-hemolytic phenotype. Based on these criteria, the isolate selected for further probiotic safety and functional characterization was preserved in MRS broth containing 20% (v/v) sterile glycerol and stored at −80 °C in cryovials until further analyses. The bacterial species used as indicator microorganisms in the antimicrobial assays included *Listeria monocytogenes* CECT 934, obtained from the Spanish Type Culture Collection, and *S. suis* LSS-Ss1602, *S. hyicus* LSS-Shyi1301, *E. faecalis* LSS-Ef1701, and *Escherichia coli* LSS-Ec1201, obtained from the bacterial collection of the Swine Health Laboratory, Faculty of Veterinary Medicine and Animal Science, University of São Paulo (FMVZ-USP). These strains were cultivated in brain heart infusion (BHI) broth at 37 °C. The identity of the selected isolate and all indicator strains was confirmed by MALDI-TOF mass spectrometry, and additional details are provided in Table 1.

**Table 1:** Field isolates of pathogenic bacteria from pigs in Brazil, used as bioindicators.

Pathogenic bacterium	Isolation site	Clinical case	Pig category	State^a^	Year
*Streptococcus suis*	Lung	Pneumonia	Nursery piglet	SP	2016
*Staphylococcus hyicus*	Skin	Pyoderma	Finisher pig	SC	2013
*Enterococcus faecalis*	Spleen	Septicemia	Sow	SP	2017
*Escherichia coli*	Feces	Enteritis	Pre-weaning piglet	RS	2012

a SP, São Paulo; SC, Santa Catarina; RS, Rio Grande do Sul.

### 2.3 Evaluation of antimicrobial activity

LAB isolates were grown in 2 mL of MRS broth under the conditions previously described. Cell-free supernatants (CFS) were obtained by centrifugation (13,000 × *g*, 10 min, 4 °C), adjusted to pH 6.0–6.5 using 1 M NaOH, and heat-treated in a water bath at 80 °C for 10 min to evaluate whether the antimicrobial activity was retained after thermal exposure. Neutralization of the CFS minimized antimicrobial effects associated with organic acid production, allowing the detection of inhibitory activity independent of medium acidification. Combined with heat treatment, this procedure served as an initial screening approach to prioritize isolates producing heat-stable antimicrobial compounds, including potential bacteriocin-like inhibitory substances.

Antimicrobial activity was evaluated against the five pathogenic bacterial species described above. Because the relationship between optical density and viable cell concentration varies among bacterial species, the optical density at 600 nm (OD₆₀₀) was previously standardized for each bioindicator strain to obtain comparable inoculum concentrations (approximately 10^8^ CFU/mL). Accordingly, bacterial cultures were adjusted to an OD₆₀₀ of 0.15–0.18 for *L. monocytogenes*; 0.20–0.25, corresponding to 3.0–4.0 × 10^8^ CFU/mL, for *S. hyicus*, *E. faecalis*, and *E. coli*; and 0.40–0.45 for *S. suis*.

Agar plates (90 mm diameter) were prepared by aseptically mixing 17.5 mL of sterile BHI agar, cooled to 45 °C, with 1% (v/v) of the corresponding bioindicator culture adjusted to the appropriate OD₆₀₀. The inoculated agar was poured into Petri dishes and allowed to solidify, after which a 7-mm diameter well was made and filled with 80 μL of the CFS from each LAB isolate. Plates were incubated at 37 °C for 24 h, and inhibition zones were measured using a digital caliper following the methodology described by Sanca et al. (2023). All experiments were performed in three independent biological replicates.


$$Ai={\left(Rt\right)}^{²}x\pi -{\left(Ro\right)}^{²}x\pi$$ (1)

$$Aa=\frac{Ai}{V}$$ (2)

The inhibition area (*Ai*, cm^2^) was calculated from the difference between the total inhibition area and the well area, and the antimicrobial activity was expressed as activity area (*Aa*, cm^2^ mL^−1^), obtained from the ratio between *Ai* and the CFS volume applied (*V*), as shown in Equations 1, 2. The antimicrobial activity of the selected isolate was further characterized by evaluating its effect on the growth kinetics of the indicator strains using a microplate assay. This complementary assay was performed to quantitatively assess the effect of the CFS on bacterial growth over time, thereby complementing the qualitative antimicrobial activity observed in the agar well diffusion assay. Only the four indicator strains that exhibited measurable inhibitory activity during the initial agar well diffusion screening (*S. suis, S. hyicus, E. faecalis,* and *L. monocytogenes*) were included in this assay. Bacterial growth inhibition was assessed by measuring OD₆₀₀ in a microplate reader (BioTek Synergy HTX, Winooski, VT, USA). The tested strains (*S. suis, S. hyicus, E. faecalis,* and *L. monocytogenes*) were adjusted to the OD₆₀₀ values described above. Each well contained 100 μL of double-strength BHI broth, 50 μL of the CFS from the selected isolate, 48 μL of sterile saline solution (0.85% NaCl, w/v), and 2 μL of the pathogen suspension (1:100, v/v). A negative control (C^−^), containing only BHI broth without bacterial inoculum, and a positive control (C^+^), containing the bacterial suspension without CFS, were included. The microplate was incubated at 37 °C for 24 h, and optical density was recorded every 15 min (Schirru et al., 2012; Sanca et al., 2023). To evaluate whether the antimicrobial activity was proteinaceous, the CFS from the selected isolate was treated with trypsin (Inlab; activity 1:250, prepared in 50 mM phosphate buffer, pH 7.0), pepsin (Inlab; activity 1:10,000, prepared in 50 mM citrate buffer, pH 2.0), or proteinase K (Inlab; activity 30 U/mg, prepared in 50 mM Tris–HCl buffer, pH 7.5) at a final concentration of 2 mg/mL and incubated at 37 °C for 1 h. The reactions were terminated by heating at 90 °C for 3 min, and the pH of the treated samples was readjusted to 6.0–6.5 before antimicrobial activity was re-evaluated using the agar well diffusion assay with *L. monocytogenes* as the indicator strain. *L. monocytogenes* was selected as the indicator strain because it exhibited the most pronounced and reproducible inhibitory response during the initial antimicrobial screening, providing a sensitive and reliable model for evaluating the effect of enzymatic treatments on the antimicrobial activity of the CFS. For each enzyme, a control containing only the corresponding enzyme in its respective buffer was included to confirm that the proteases themselves had no inhibitory effect on the indicator strain.

### 2.4 Biochemical characterization and evaluation of potential virulence factors

Catalase, gelatinase, hemolytic, and coagulase activities were evaluated as part of the biochemical characterization and assessment of potential virulence-associated traits. For the catalase test, bacterial cultures were placed on microscope slides, and a drop of 3% hydrogen peroxide (Sigma–Aldrich®, Merck KGaA, Darmstadt, Germany) was added directly onto the bacterial colony. Catalase activity was determined by the immediate formation of oxygen bubbles upon contact with hydrogen peroxide. To determine gelatinase activity, a gelatinase detection medium was prepared according to standard microbiological protocols, containing 3% (w/v) gelatin, 0.5% (w/v) peptone, 0.3% (w/v) yeast extract, and 0.3% (w/v) beef extract. Cryopreserved cultures were reactivated by overnight incubation in 4 mL of MRS broth at 37 °C. After activation, a 100-μL aliquot of the culture was inoculated into 3 mL of gelatin medium and incubated at 37 °C for 24 h under microaerophilic conditions. Subsequently, cultures were transferred to 4 °C for 1 h, and the absence of gelatin liquefaction after refrigeration was interpreted as a negative result for gelatinase activity. Hemolytic activity was assessed using Blood Agar (BA) plates supplemented with 5% sheep blood (Difco, BD, USA). Overnight cultures were streaked onto Blood Agar plates and incubated at 35 °C for 48–72 h under microaerophilic conditions. Hemolytic activity was determined based on the appearance of zones around the colonies, with clear zones indicating β-hemolysis (complete hemolysis), greenish zones indicating α-hemolysis (partial hemolysis), and no visible changes indicating γ-hemolysis (non-hemolytic reaction). For the coagulase test, 500 μL of rabbit plasma (COAGU-PLASMA, Laborclin, Brazil) was mixed with 500 μL of an overnight bacterial culture, and the tubes were incubated at 37 °C for 24 h under microaerophilic conditions. *Staphylococcus aureus* CECT 239 was used as a positive control for coagulase activity. Plasma coagulation (solidification) indicated a positive coagulase reaction, whereas the absence of coagulation was interpreted as a negative result.

### 2.5 Antimicrobial susceptibility testing

The antimicrobial susceptibility of the LBM-M3D isolate was evaluated by disk diffusion and broth microdilution methods. For the disk diffusion assay, susceptibility was determined according to the Clinical and Laboratory Standards Institute guidelines [Clinical Laboratory Standards Institute (CLSI), 2024a], with modifications for LAB as described by Charteris et al. (1998). Petri dishes containing 15 mL of MRS agar were prepared, and LAB suspensions (approximately 10^6^–10^7^ CFU/mL) were mixed with 4 mL of 0.6% (w/v) MRS agar, from which 40 μL was spread onto each plate. Antibiotic discs (Laborclin, Brazil) were placed on the agar, with a maximum of four discs per plate: rifampicin (RIF, 5 μg), erythromycin (ERY, 15 μg), tetracycline (TET, 30 μg), chloramphenicol (CHL, 30 μg), clindamycin (CLI, 2 μg), streptomycin (STR, 10 μg), ampicillin (AMP, 2 μg), and vancomycin (VAN, 5 μg). Plates were left at room temperature for 15 min to allow antibiotic diffusion and subsequently incubated at 37 °C under microaerophilic conditions for 24 h. Inhibition zones were measured in millimeters using a digital caliper and interpreted as susceptible or resistant according to the breakpoints proposed by Charteris et al. (1998).

To complement the disk diffusion assay, the minimum inhibitory concentrations (MICs) of ampicillin, clindamycin, chloramphenicol, erythromycin, gentamicin, kanamycin, streptomycin, tetracycline, and vancomycin were determined using the broth microdilution method following CLSI guidelines [Clinical Laboratory Standards Institute (CLSI), 2024b]. The CLSI recommendations were used exclusively to standardize the experimental procedures. However, antibiotic selection and interpretation of MIC values were based on the recommendations of the European Food Safety Authority [EFSA Panel on Additives and Products or Substances used in Animal Feed (FEEDAP) et al., 2018], which provide epidemiological cutoff values (ECOFFs) for the safety assessment of *Enterococcus* spp. intended for use in the food and feed chain.

Working solutions were prepared by serial twofold dilution of each antibiotic and dispensed into microtiter plates. Each well was inoculated with 5 × 10^5^ CFU/mL of bacterial suspension prepared in cation-adjusted Mueller–Hinton broth (CAMHB) supplemented with 10% MRS medium to support LAB growth (Klare et al., 2007). Plates were incubated at 37 °C under microaerophilic conditions for 12–18 h. Minimum inhibitory concentration values were interpreted as susceptible or resistant according to the microbiological cutoff values established by the EFSA FEEDAP Panel for *Enterococcus* spp. Quality control was performed using the reference strains *E. coli* ATCC 25922 and *Staphylococcus aureus* ATCC 29213, following CLSI recommendations. The MIC values obtained for both reference strains were within the corresponding CLSI quality control ranges, confirming the reliability of the antimicrobial susceptibility assay.

### 2.6 *In vitro* tolerance to acidic pH and bile salts

The selected LAB isolate was evaluated for its tolerance to bile salts and different acidic and alkaline pH conditions under *in vitro* stress conditions. The incubation period (20 h) was selected to evaluate the ability of the isolate to remain viable following prolonged exposure to acid and bile salt stress rather than to simulate physiological gastrointestinal transit.

Initially, the isolate was inoculated into 10 mL of MRS broth and incubated at 37 °C under microaerophilic conditions for 20 h. Cells were harvested by centrifugation (13,000 × *g*, 10 min, 4 °C), washed twice with sterile phosphate-buffered saline (PBS 1×), and resuspended to an OD₆₀₀ of 1.0. The bacterial suspension was previously standardized so that an OD₆₀₀ of 1.0 corresponded to 3–4 × 10^8^ CFU/mL and was used to normalize the inoculum concentration throughout all experiments.

For the bile salt tolerance assay, 1 mL of the standardized bacterial suspension was transferred to 5 mL of MRS broth supplemented with 0.1, 0.2, 0.3, 0.4%, or 0.5% (w/v) bile salts and incubated at 37 °C under microaerophilic conditions for 20 h. For the pH tolerance assay, 1 mL of the same standardized suspension was transferred to 5 mL of MRS broth adjusted to pH 2.0, 3.0, 4.0, 6.0, or 8.0 using 1 M HCl or NaOH and incubated under identical conditions. Following incubation, samples from both assays were serially diluted (10^−1^ to 10^−7^), plated onto MRS agar, and incubated at 37 °C for 40 h. Bacterial survival under these stress conditions was determined exclusively by viable cell counts and expressed as colony-forming units per milliliter (CFU/mL).

To complement the survival assays, the effect of pH on bacterial growth was further evaluated by cultivating the selected isolate in MRS broth adjusted to pH 3.0, 4.0, 6.0, and 8.0 using the same standardized inoculum. This assay was designed to characterize bacterial growth dynamics rather than bacterial survival under stress conditions. Growth was monitored by measuring the optical density at 600 nm (OD₆₀₀) every 15 min using a microplate reader (BioTek Synergy HTX, Winooski, VT, USA). Each well contained 190 μL of MRS broth adjusted to the corresponding pH and 10 μL of the bacterial suspension, resulting in a final volume of 200 μL and an initial OD₆₀₀ of 0.1. Commercial MRS broth (pH 6.5) was used as the positive control. Microplates were incubated at 37 °C for 22 h, and all experiments were performed in triplicate. The relative difference in bacterial growth at each time point was calculated as:


$$\mathrm{Relative Difference}=\left({\mathrm{OD}}_{p}H-\mathrm{ODcontrol}\right)/\mathrm{ODcontrol}$$

where OD_p_H represents the optical density measured at the evaluated pH and ODcontrol corresponds to the optical density of the control culture grown in MRS broth at pH 6.5.

### 2.7 Partial purification of antimicrobial molecule

#### 2.7.1 Tangential flow filtration for CFS fractionation

An 8-mL pre-inoculum of the selected LAB isolate was prepared from a cryopreserved stock and incubated at 37 °C for 24 h under microaerophilic conditions. The pre-inoculum was subsequently transferred to 400 mL of MRS broth and incubated at 37 °C for 19 h with agitation at 130 rpm. The cell-free supernatant (CFS) was obtained as previously described. Tangential flow filtration was performed using a 2 kDa molecular weight cutoff membrane (Sartorius AG, Göttingen, Germany) at a flow rate of 12 mL/min until the retentate volume reached approximately 10% of the initial volume. The resulting retentate was subsequently evaluated for antimicrobial activity against *L. monocytogenes*.

#### 2.7.2 Cation exchange chromatography

The retentate obtained by tangential flow filtration was loaded onto a cation-exchange chromatography column packed with 40 mL of SP Sepharose resin (Cytiva, Marlborough, MA, USA), previously equilibrated with 300 mL of 20 mM sodium acetate buffer (pH 4.4). The column was washed with 100 mL of the same buffer supplemented with 100 mM NaCl, and the bound compounds were subsequently eluted with 300 mL of 20 mM sodium acetate buffer (pH 4.4) containing 1 M NaCl. The eluted fraction was lyophilized for 3–5 days under vacuum (−80 °C, 0.09 mT) using an L101 bench-top lyophilizer (Liobras, São Carlos, SP, Brazil).

#### 2.7.3 Solid-phase extraction chromatography

The fraction exhibiting antimicrobial activity was further purified using solid-phase extraction (SPE) chromatography with a C18 cartridge (DSC-18 SPE, Discovery®). The SPE cartridge was first equilibrated with 10 mL of water containing 0.1% (v/v) trifluoroacetic acid (TFA). After loading the supernatant samples, elution was performed consecutively by applying 10 mL of 50% acetonitrile and 10 mL of 70% acetonitrile, both containing 0.1% (v/v) TFA. Organic solvents from each fraction were removed using a vacuum centrifuge, and the pH was adjusted to 6.0–6.5. The antimicrobial activity of the samples was then evaluated against *L. monocytogenes*. Finally, all fractions that presented antimicrobial activity were freeze-dried under vacuum (−80 °C, 0.09 mT) using the L101 bench-top lyophilizer (Liobras, Brazil).

#### 2.7.4 High-performance liquid chromatography (HPLC)

Samples were injected into a high-performance liquid chromatograph (Agilent Technologies, Santa Clara, California, USA) equipped with two pumps, an analytical C18 column (250 × 4.6 mm, 5 μm), and a UV–VIS detector set at 220 and 280 nm. The injection volume was 50 μL per run. Peptides were separated using a linear gradient elution, transitioning from 99% mobile phase A (99.9% H₂O/0.1% TFA) to 99% mobile phase B (99.9% acetonitrile/0.1% TFA) at a flow rate of 1 mL/min over 52 min. The bioactive fraction was collected between 30.9 and 36.5 min, which corresponds to an acetonitrile concentration of 46.3–52.6%. Chromatographic fractions were collected based on UV absorbance at 220 nm, and the active fraction corresponding to the 30.9–36.5 min interval was selected for further analysis. The bioactivity of each fraction was assessed following the methodology described using *L. monocytogenes* as the bioindicator bacterium.

### 2.8 Whole-genome sequencing, assembly, and annotation

Whole-Genome Sequencing, Assembly, and Annotation Genomic DNA was extracted from pure bacterial cultures utilizing the PureLink Genomic DNA Mini Kit (Invitrogen, Thermo Fisher Scientific, USA) in accordance with the manufacturer’s protocol. To ensure sample integrity, the concentration and purity of the extracted DNA were quantitatively assessed via spectrophotometry using a NanoDrop One instrument and via fluorometry using a Qubit 2.0 fluorometer (Thermo Fisher Scientific). Subsequently, genomic libraries were prepared following the standard protocol for PacBio HiFi technology, and whole-genome sequencing was performed on the PacBio Revio platform at the Arizona Genomics Institute (AGI). Sequencing performance was evaluated via SMRT Link Analysis, yielding a high-quality dataset characterized by a median HiFi read quality of Q42, a barcode quality of 97.3, and a HiFi yield of 1,136,314 bp. The mean polymerase read length achieved was 141,112 bp, generating a mean HiFi read length of 9,047 bp.

*De novo* genome assembly was executed using Hifiasm (v0.19.8-r603) (Cheng et al., 2021), followed by contig reorientation utilizing DNAapler (v0.7.0) (Bouras et al., 2024). Circular genome representations were generated using the GenoVi pipeline (v0.4.3) (Cumsille et al., 2023). The overall quality of the assembly was rigorously evaluated using the QUAST, CheckM, and BUSCO pipelines, as outlined in Dos Anjos Almeida et al. (2025). The final assembly achieved an N50 of 2,450,530 bp, an L50 of 2, and a mean sequencing depth of 109x. This combination of deep coverage and long-read capability allowed for the complete, unfragmented resolution of both the circular chromosome and the 160 kb plasmid, seamlessly bridging repetitive insertion sequences. Finally, structural and functional annotation was carried out utilizing the NCBI Prokaryotic Genome Annotation Pipeline (PGAP).

Taxonomic classification was confirmed via Average Nucleotide Identity (ANI) calculated using ANIclustermap (v1.4.0)^1^ to compare the LBM-M3D strain with fourteen *Enterococcus lactis* and fourteen *E. faecium* reference genomes (Supplementary Table SM1), allowing for the robust evaluation of genetic similarities and species boundaries.

The genomic screening for antimicrobial resistance (AMR) determinants, plasmid replicons, and putative virulence factors was conducted using the ABRICATE pipeline (v1.0.0).^2^ To ensure high-confidence alignments, stringent cut-off thresholds of 80% sequence identity and 80% coverage were applied across all searches. The screening for antimicrobial resistance and related elements was queried against the CARD, ResFinder, ARG-ANNOT, MEGARES, and PlasmidFinder databases. Additionally, the characterization of virulence factors was performed by querying the assemblies against the VFDB, Victors, ecoli_vf, and ecoh databases. Following EFSA guidelines [EFSA Panel on Biological Hazards (BIOHAZ) et al., 2023, 2025], an AMR-associated gene was considered putatively intrinsic when it was chromosomally located in LBM-M3D and conserved across publicly available *E. lactis* genomes included in the comparative dataset. Conversely, genes absent from the comparative chromosomal dataset or associated with plasmid regions and mobile genetic elements were considered potentially acquired.

Biosynthetic gene clusters (BGCs) potentially involved in antimicrobial activity were identified using BAGEL (v4.0) (van Heel et al., 2018) and antiSMASH (v7.1) (Blin et al., 2023). In parallel, the potential to express antimicrobial peptides was evaluated through *de novo* identification using AmpGram (Burdukiewicz et al., 2020), AMP Scanner v2 (Veltri et al., 2018), AMPlify (Li et al., 2022), amPEPpy (Lawrence et al., 2021), and marcel (Santos-Júnior et al., 2020). All figures were edited using Inkscape.^3^

### 2.9 Statistical analysis

All experiments were performed using three independent biological replicates (*n* = 3), and numerical data were expressed as the mean ± standard deviation (SD). For antimicrobial activity, a one-sample *t*-test was used to determine if the activity of each strain was significantly different from zero. To evaluate tolerance to different pH levels and bile salt concentrations, a one-way analysis of variance (ANOVA) was performed, with a Dunnett’s *post-hoc* test to compare each condition to its respective control. In all cases, the 95% confidence intervals (95% CI) and exact *p*-values were reported, with a *p*-value of <0.05 considered statistically significant.

## 3 Results

### 3.1 Isolation, selection and identification of the LBM-M3D isolate

A total of 30 morphologically distinct LAB isolates were recovered from the cecal contents of three healthy pigs, with 10 isolates obtained from each animal. For each animal, five isolates were recovered from plates incubated under aerobic conditions and five from plates incubated under microaerophilic conditions. All isolates were screened for antimicrobial activity against *L. monocytogenes*, *E. faecalis*, *S. hyicus*, *S. suis*, and *E. coli* using the agar well diffusion assay. Of the 30 isolates evaluated, 21 did not exhibit detectable inhibitory activity against any of the indicator strains. Seven isolates inhibited exclusively *L. monocytogenes*, whereas two isolates displayed a broader antimicrobial spectrum, inhibiting *L. monocytogenes*, *E. faecalis*, *S. hyicus*, and *S. suis*. As part of the safety screening, these two isolates were subsequently evaluated for hemolytic activity. One isolate, recovered from animal 1, exhibited β-hemolysis and was excluded from further analyses. The remaining non-hemolytic isolate, recovered from plates incubated under microaerophilic conditions from animal 3, was selected for subsequent phenotypic, genomic, and functional characterization and was designated LBM-M3D. The antimicrobial activity of the neutralized and heat-treated cell-free supernatant (CFS) produced by LBM-M3D is summarized in Table 2.

**Table 2:** Antimicrobial activity of neutralized and heat-treated LBM-M3D cell-free supernatant (CFS) against Gram-positive and Gram-negative bacteria.

Pathogenic strains	Area of inhibition (cm^2^)	Diameter of inhibition halo (mm)	Antimicrobial activity (*Aa*)	*p*-value	95% CI
*Staphylococcus hyicus*	1.44 ± 0.22	15.24 ± 1.74	18.0^*^	0.0010	(14.5, 19.44)
*Streptococcus suis*	0.78 ± 0.27	12.20 ± 1.47	9.8^*^	0.0086	(5.13, 12.02)
*Enterococcus faecalis*	1.66 ± 0.20	16.14 ± 0.79	20.8^*^	0.0001	(19.86, 21.11)
*Listeria monocytogenes*	4.34 ± 0.07	24.52 ± 1.03	54.2^*^	0.0005	(47.42, 57.51)
*Escherichia coli*	0	0	0	Not applicable	Not applicable

The inhibitory effect was evaluated using CFS adjusted to pH 6.0–6.5 (neutralized) and treated at 80 °C for 10 min (heat-treated). Data are presented as the mean ± standard deviation (*n* = 3 independent biological replicates).

^*^p-values represent the results of a one-sample *t*-test comparing the observed inhibition area against a theoretical null value of zero, indicating that the recorded inhibition areas were significantly different from zero (*p* < 0.05).

The selected isolate, designated LBM-M3D, was initially identified by Matrix-Assisted Laser Desorption/Ionization Time-of-Flight Mass Spectrometry (MALDI-TOF MS) using the Autoflex Speed Smartbeam system (Bruker Daltonics, Germany). MALDI-TOF MS analysis yielded a reliable species-level identification score, classifying the isolate as *E. faecium*. However, whole-genome sequencing and average nucleotide identity (ANI) analysis were subsequently performed to establish its definitive taxonomic classification.

### 3.2 Evaluation of LBM-M3D’s probiotic-associated properties

#### 3.2.1 Biochemical characterization and evaluation of potential virulence factors

Several biochemical traits were evaluated in the LBM-M3D strain, including its potential to produce enzymes such as catalase, coagulase, gelatinase, and hemolysin, which are considered potential virulence factors. However, LBM-M3D tested negative for all these enzymatic activities. The presence of a γ-hemolysis reaction, indicative of a lack of hemolytic activity, further supports this finding.

#### 3.2.2 Antibiotic resistance

The antimicrobial susceptibility of the LBM-M3D strain was evaluated against a total of 10 antibiotics using two complementary methods. Eight antibiotics were tested by disk diffusion, including rifampicin, while nine were assessed by broth microdilution. For the antibiotics evaluated by microdilution, the LBM-M3D strain exhibited MIC values below the corresponding microbiological cut-offs, whereas rifampicin assessed exclusively by disk diffusion was classified as susceptible (Table 3).

**Table 3:** Antimicrobial susceptibility of the LBM-M3D strain determined by disk diffusion and broth microdilution methods.

Antibiotic	Antimicrobial disk diffusion					Minimal inhibitory concentration (MIC)	
	Disc potencies (μg)	Disk diffusion (mm) classification			Resistance profile	EFSA MIC breakpoints (μg/mL)	Resistance profile
		R	I	S			
Rifampicin	5	≤14	15–17	≥18	28,2 (S)	ND	ND
Erythromycin	15	≤13	14–17	≥18	11,6 (R)	4	32 (R)
Tetracycline	30	≤14	15–18	≥19	31,2 (S)	4	0,25 (S)
Chloramphenicol	30	≤13	14–17	≥18	25,7 (S)	16	2 (S)
Clindamycin	2	≤8	09–11	≥12	27,1 (S)	4	0,5 (S)
Streptomycin	10	≤11	12–14	≥15	15,6 (S)	128	128 (S)
Kanamycin	ND	ND	ND	ND	ND	1,024	1,024 (S)
Gentamicin	ND	ND	ND	ND	ND	32	16 (S)
Ampicillin	2	≤12	13–15	≥16	20,6 (S)	2	1 (S)
Vancomycin	5	≤14	15–16	≥17	20,7 (S)	4	1 (S)

ND, not determined; S, susceptible; MS, moderately; R, resistant.

Susceptibility to the antibiotics was classified as Susceptible (S), Intermediate (I), or Resistant (R) based on the microbiological cutoff values from EFSA and CLSI guidelines. ND indicates that the disk diffusion test was not performed.

#### 3.2.3 Viability of LBM-M3D under different pH and bile salt conditions

The LBM-M3D strain was tested under different pH conditions and bile salt concentrations (Table 4). After 20 h of exposure, bile salt tolerance tests (*n* = 3) showed that the viable count increased to (1.0 ± 0.25) × 10^9^ CFU/mL (9.00 ± 0.10 Log₁₀ CFU/mL; 217.4% relative survival) at 0.1% bile salts, compared with the control (4.6 ± 0.35 × 10^8^ CFU/mL; 8.66 ± 0.03 Log₁₀ CFU/mL; *p* < 0.0001). In contrast, viable counts decreased to (4.0 ± 1.00) × 10^7^, (4.0 ± 1.10) × 10^7^, (3.3 ± 0.25) × 10^7^, and (2.8 ± 0.72) × 10^7^ CFU/mL, corresponding to 7.60 ± 0.11, 7.60 ± 0.12, 7.52 ± 0.03, and 7.45 ± 0.11 Log₁₀ CFU/mL, with 8.7, 8.7, 7.2, and 6.1% relative survival at bile salt concentrations of 0.2, 0.3, 0.4, and 0.5%, respectively (*p* < 0.05 for all concentrations). Although the increase observed at 0.1% bile salts was statistically significant, the difference relative to the control was less than 0.5 Log₁₀ CFU/mL. Since variations below one logarithmic unit remain within the same order of magnitude in microbiological analyses, this result is more consistent with the strain’s high physiological stability and tolerance to mild bile stress than with a biologically meaningful enhancement of bacterial growth.

**Table 4:** Survival of the potentially probiotic LBM-M3D strain after 20 h of exposure to different pH conditions and bile salt concentrations, expressed as CFU/mL, relative survival (%), and Log_10_ CFU/mL.

Condition	CFU/mL (Mean ± SD)	Relative survival (%)	Log_10_ CFU/mL	*p*-value
Bile salt				
0.5%	(2.8 ± 0.72) × 10^7^	6.1%	7.45 ± 0.11	<0.0001*
0.4%	(3.3 ± 0.25) × 10^7^	7.2%	7.52 ± 0.03	0.0028*
0.3%	(4.0 ± 1.10) × 10^7^	8.7%	7.60 ± 0.12	<0.0001*
0.2%	(4.0 ± 1.00) × 10^7^	8.7%	7.60 ± 0.11	0.0039*
0.1%	(1.0 ± 0.25) × 10^9^	217.4%	9.00 ± 0.1	<0.0001*
pH				
8.0	(4.8 ± 0.62) × 10^8^	104.3%	8.68 ± 0.05	>0.9999
6.0	(1.4 ± 0.16) × 10^9^	304.3%	9.15 ± 0.05	<0.0001*
4.0	(1.7 ± 0.24) × 10^8^	36.9%	8.23 ± 0.06	<0.0001*
3.0	(1.0 ± 0.35) × 10^4^	0.002%	4.00 ± 0.15	<0.0001*
2.0	<10		< 1.00	0.0014*
Control (pH 6.5, 0.0% bile salts)	(4.6 ± 0.35) × 10^8^	100%	8.66 ± 0.03	–

Values represent the mean ± standard deviation (SD) of three independent experiments (*n* = 3). Viability is expressed as CFU/mL, Log₁₀ CFU/mL, and relative survival (%). Relative survival was calculated using the control condition (pH 6.5/0% bile salts) as the reference. Statistical significance was determined by one-way ANOVA followed by Dunnett’s *post hoc* test against the corresponding control group. An asterisk (*) indicates a statistically significant difference (*p* < 0.05). <10 CFU/mL (<1.00 Log₁₀ CFU/mL) represents the limit of detection.

The analysis of LBM-M3D tolerance under different pH conditions (*n* = 3) showed that the viable count increased to (1.4 ± 0.16) × 10^9^ CFU/mL (9.15 ± 0.05 Log₁₀ CFU/mL; 304.3% relative survival) at pH 6.0, significantly higher than the control (*p* < 0.0001). At pH 8.0, the viable count was (4.8 ± 0.62) × 10^8^ CFU/mL (8.68 ± 0.05 Log₁₀ CFU/mL; 104.3% relative survival), with no significant difference compared with the control (*p* > 0.9999), whereas at pH 4.0 it decreased to (1.7 ± 0.24) × 10^8^ CFU/mL (8.23 ± 0.06 Log₁₀ CFU/mL; 36.9% relative survival) (*p* < 0.0001). At pH 3.0, viability was markedly reduced to (1.0 ± 0.35) × 10^4^ CFU/mL (4.00 ± 0.15 Log₁₀ CFU/mL; 0.002% relative survival) (*p* < 0.0001), whereas no colonies were detected at pH 2.0 (<10 CFU/mL; <1.00 Log₁₀ CFU/mL).

**Figure 1:**
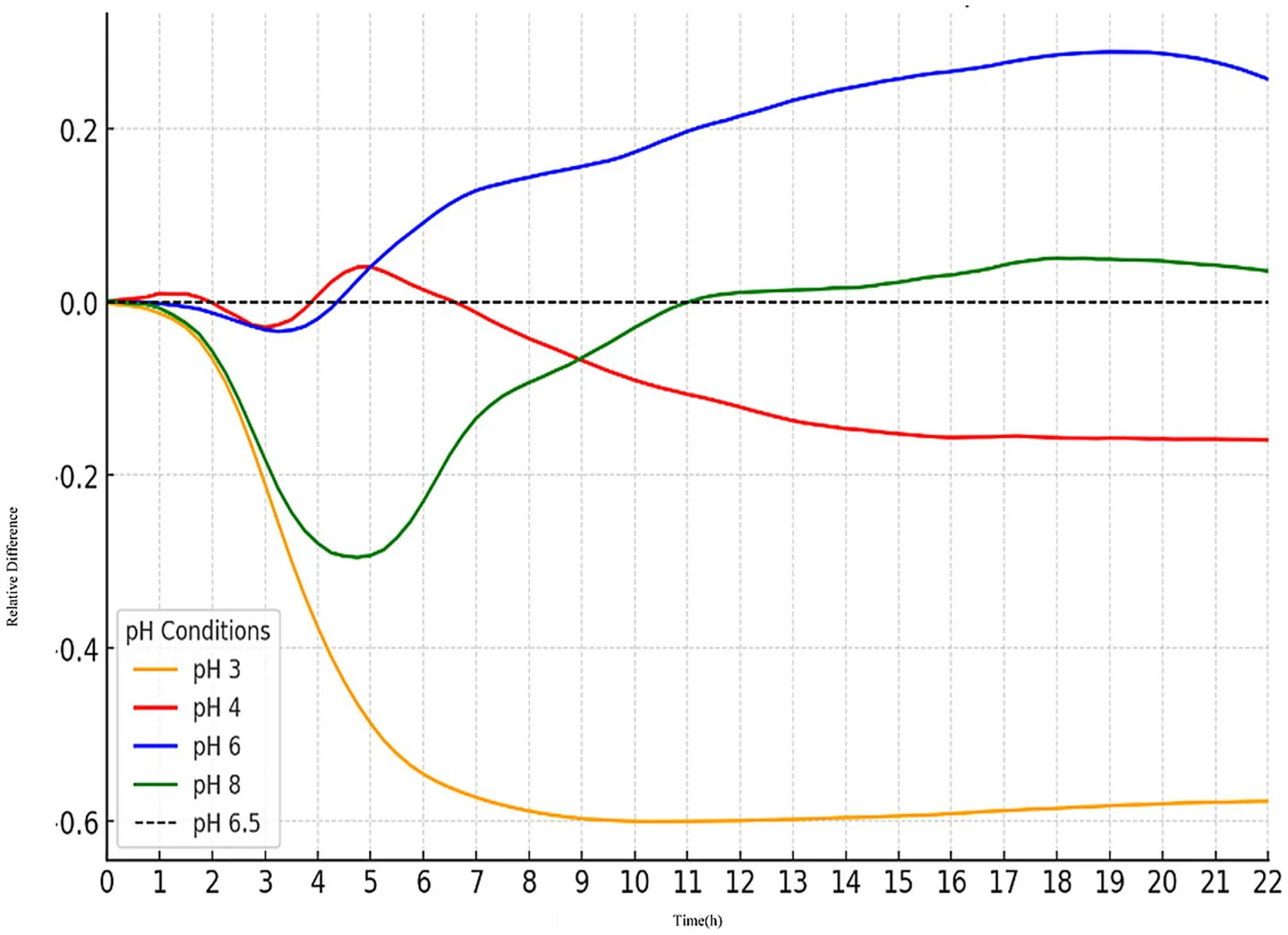
Relative difference in the growth of strain LBM-M3D in MRS medium adjusted to different pH values (3, 4, 6 and 8) over 22 h. (----): The black dashed line represents the control pH 6.5 (MRS without pH adjustment). The Y-axis represents the relative difference in growth, meaning that values above zero indicate higher growth compared to the control, while values below zero indicate reduced growth.

Growth kinetics monitored by automated OD₆₀₀ measurements throughout the 20-h incubation period (Figure 1) were consistent with the endpoint viable counts presented in Table 4, showing the highest relative growth at pH 6.0, a slight increase at pH 8.0, and marked inhibition at pH 3.0 and pH 4.0. Overall, these findings indicate that LBM-M3D maintained high viability under near-neutral and mildly acidic conditions, with optimal performance around pH 6.0, whereas increasingly acidic environments markedly impaired bacterial growth and survival.

### 3.3 Characterization of the CFS from strain LBM-M3D

#### 3.3.1 Peptide nature

The effect of proteolytic enzymes on the antimicrobial activity of LBM-M3D’s CFS was evaluated (Table 5). Treatment with trypsin completely abolished the inhibitory effect, while pepsin and proteinase K reduced the inhibition zone by more than 40% relative to its initial size (1.07 cm). These results suggest that the antimicrobial activity of LBM-M3D’s CFS is associated with a proteinaceous or peptide-like component, likely related to bacteriocinogenic activity.

**Table 5:** Effect of enzymatic treatment on the antimicrobial activity of LBM-M3D CFS against *Listeria monocytogenes*.

Proteases	Strain LBM-M3D^a^
Cell free supernatant (control)	1.07 ± 0.02 cm
Trypsin	0.0 cm
Pepsin	0.19 ± 0.04 cm
Proteinase K	0.33 ± 0.16 cm

a Values represent mean ± standard deviation (SD) from three independent replicates.

Measurements in centimeters (cm).

#### 3.3.2 Kinetics and inhibition of pathogens by LBM-M3D CFS

**Figure 2:**
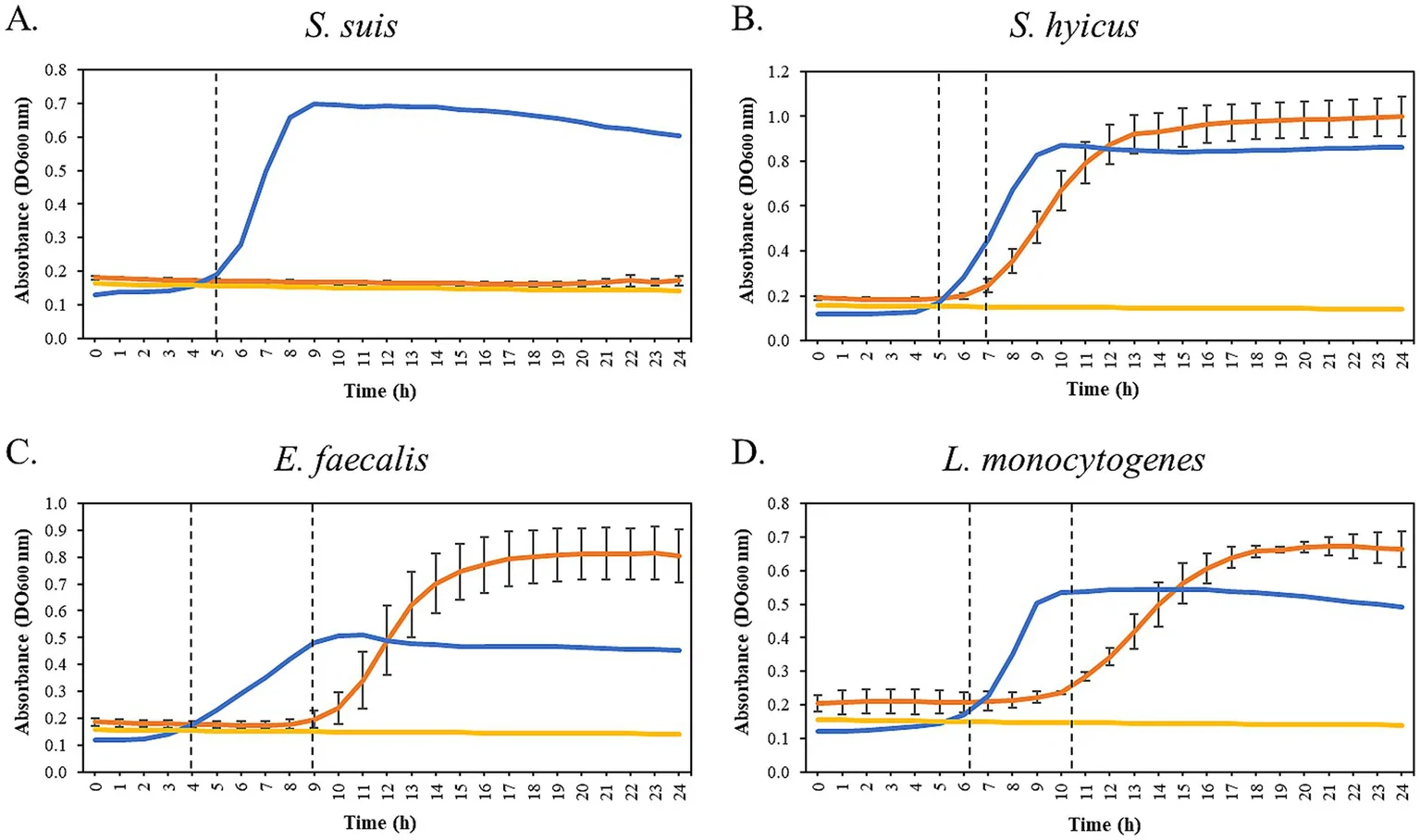
Growth kinetics of pathogenic strains after 24 h of exposure to LBM-M3D CFS. The orange line represents the growth of the pathogenic strain in the presence of CFS (treated), the blue line represents its growth in the absence of CFS (positive control, C+), and the yellow line represents the negative control (C-), which contains CFS and culture medium but no pathogenic strain. The tested pathogens are represented as follows: **(A)** *Streptococcus suis*; **(B)** *Staphylococcus hyicus*; **(C)** *Enterococcus faecalis*; and **(D)** *Listeria monocytogenes*.

To evaluate the inhibitory effect of LBM-M3D’s CFS on susceptible pathogens, growth kinetics were monitored over 24 h (Figure 2). Of the four pathogens tested, three showed delayed growth in the presence of CFS, as evidenced by a delay in reaching the exponential phase. *Staphylococcus hyicus* (Figure 2B) was the least affected, with only a 2-h delay relative to the positive control (C+). *Enterococcus faecalis* (Figure 2C) and *L. monocytogenes* (Figure 2D) exhibited longer delays of 4 and 5 h, respectively. In contrast, *S. suis* exhibited complete suppression of detectable growth by OD₆₀₀ over the 24-h period. The absence of an exponential phase, which closely mirrored the negative control (C–), indicates strong growth inhibition under the tested conditions. The lowest antimicrobial activity in the agar diffusion assay occurred against *S. hyicus* (18 *Aa*), whereas *E. faecalis* and *L. monocytogenes* reached higher values (20.8 *Aa* and 54.2 *Aa*, respectively). However, for *S. suis*, the lower *Aa* value observed in agar diffusion did not directly reflect the strong growth suppression detected in liquid culture, suggesting that agar diffusion activity and growth-kinetic inhibition may not be directly comparable across pathogen species.

### 3.4 High-performance liquid chromatography (HPLC) and MALDI-TOF analysis of LBM-M3D CFS

**Figure 3:**
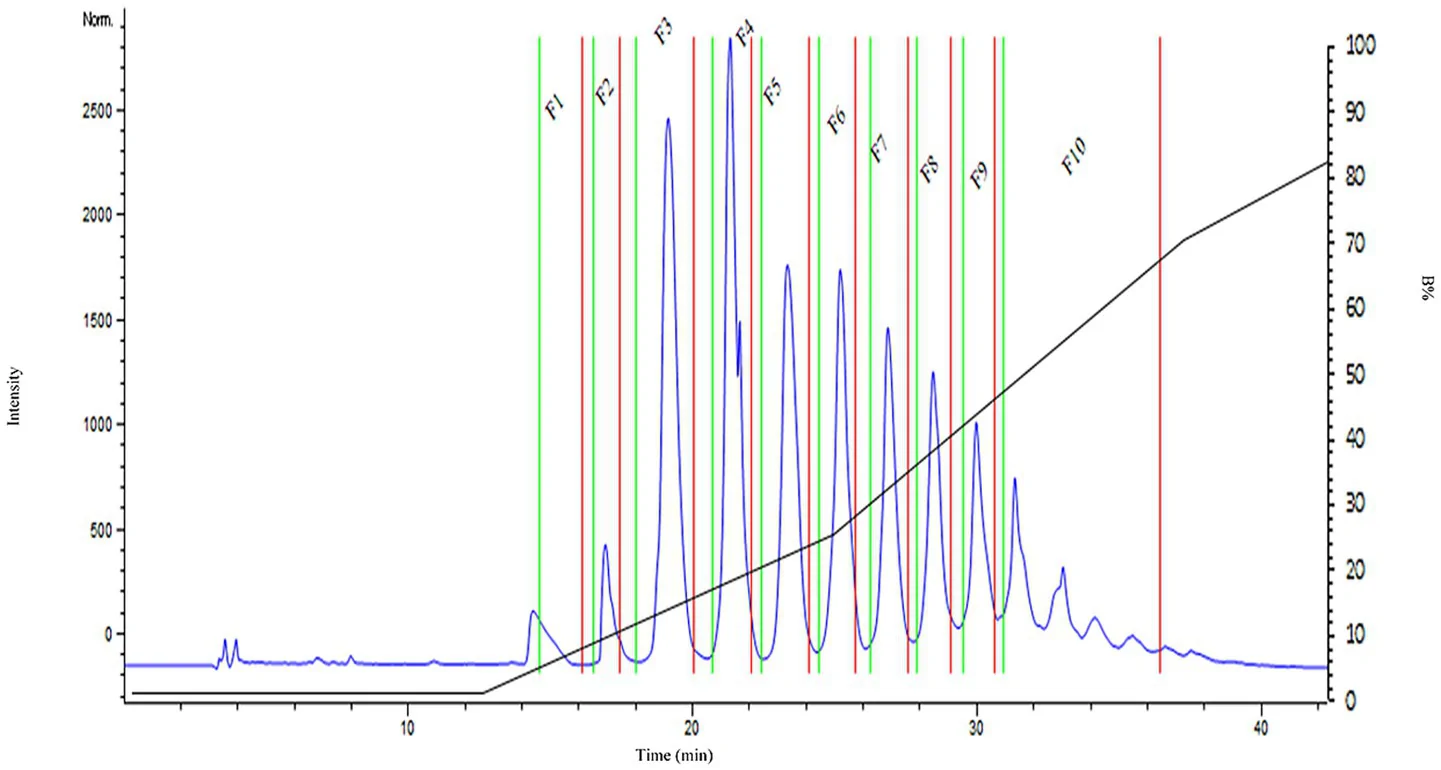
HPLC chromatogram of LBM-M3D CFS (A) chromatographic profile of the separation of the LBM-M3D cell-free supernatant (CFS) by HPLC, showing the collection of ten fractions. The green lines indicate the start, and the red lines indicate the end of each fraction. The elution gradient, representing the percentage variation of solvent B (acetonitrile) over time, is the black line. The active fraction (F10), identified as containing antimicrobial activity, is annotated on the chromatogram.

After purifying LBM-M3D’s CFS by tangential flow filtration (2 kDa membrane), ion exchange chromatography (SP-Sepharose beads), and solid-phase chromatography (C18 column), the bioactivity was monitored at each step against *L. monocytogenes*. The final purified sample was analyzed by HPLC (Figure 3), and ten peaks were collected. Of these, only Fraction 10, which eluted at 30.9–36.5 min with an acetonitrile concentration of 46.3–52.6%, exhibited antimicrobial activity.

To confirm the proteinaceous nature of Fraction 10, it was enzymatically digested using trypsin, pepsin, and proteinase K, followed by an antimicrobial activity assay against *L. monocytogenes*. As shown in Table 6, protease treatment resulted in complete loss of inhibition, whereas the untreated control exhibited a 0.94 cm inhibitory halo against *L. monocytogenes*. These results support the involvement of a proteinaceous or peptide-associated component in the antimicrobial activity of Fraction 10, consistent with bacteriocin-like activity. Although the identity of the active antimicrobial molecule was not established in the present study.

**Table 6:** Effect of protease treatment on the antimicrobial activity of fraction 10 against *Listeria monocytogenes*.

Protease treatment	Inhibition zone (cm)
Control (untreated)	0.94 ± 0.03 cm
Trypsin	0.0 cm
Pepsin	0.0 cm
Proteinase K	0.0 cm

Measurements in centimeters (cm).

### 3.5 Genomic characterization and probiotic-associated features of LBM-M3D

**Figure 4:**
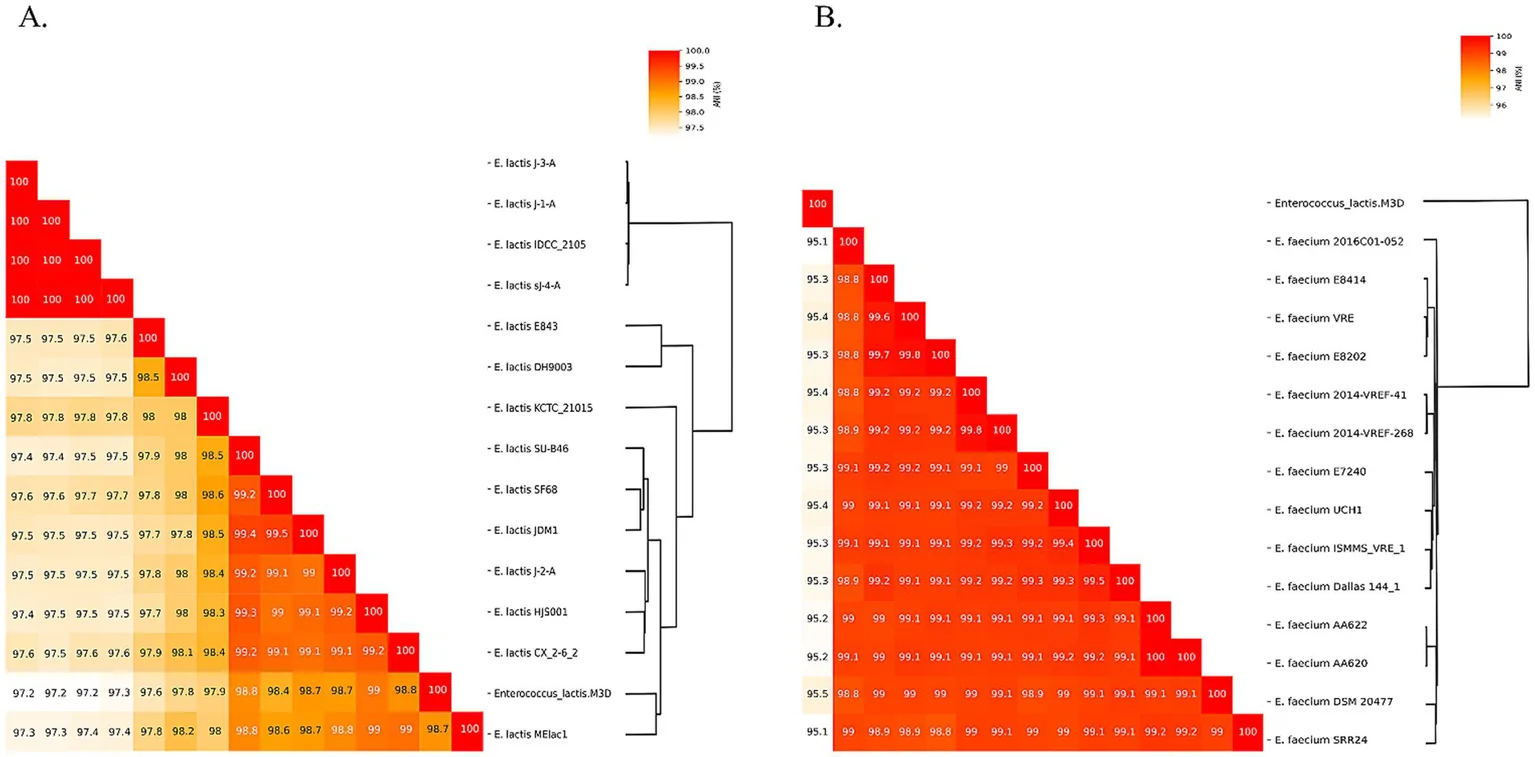
Heatmap showing the comparative ANI values between the genome of LBM-M3D and 14 complete *E. lactis* genomes **(A)** as well as 14 complete *E. faecium* genomes **(B)**.

The isolate LBM-M3D, obtained from the cecal content of adult pigs, was sequenced and classified as *Enterococcus lactis*. A heatmap of ANI (Average Nucleotide Identity) values was generated using sequences from closely related species to the LBM-M3D strain. The results confirmed its classification as *E. lactis*, with an ANI value >97.8% when compared to the reference genome *E. lactis* DH9003 (Figure 4A). Additionally, *E. faecium* exhibited ANI values <95.5%, showing significant genetic differentiation from LBM-M3D (Figure 4B).

**Figure 5:**
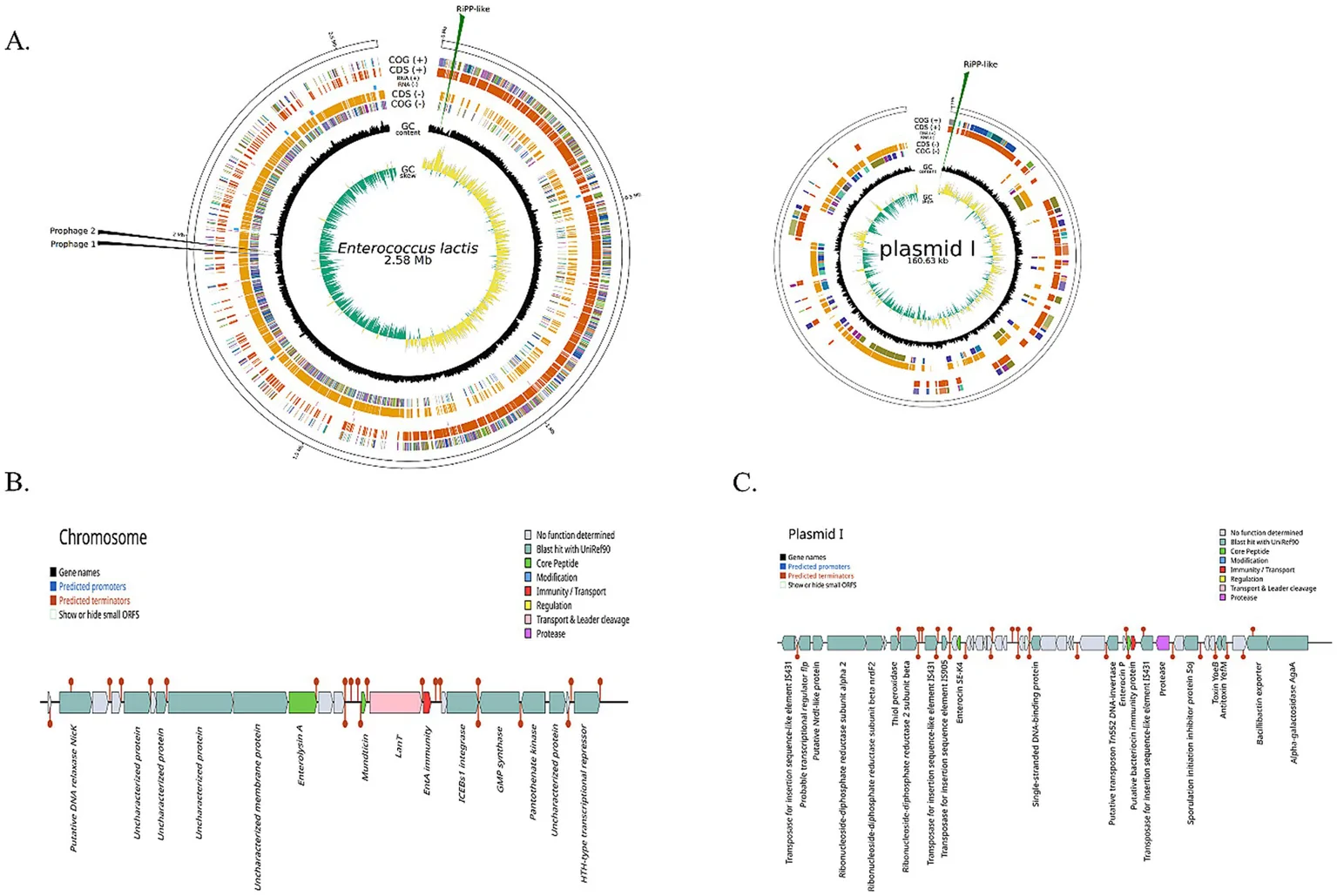
Circular representation of the genetic features in the LBM-M3D genome and its plasmids. The map was generated using the GenoVi pipeline (v0.4.3). **(A)** The main circular map illustrates the chromosome (*Enterococcus lactis*, 2.58 Mb) and plasmid I (160.63 Kb). The circles, from outermost to innermost, represent: The genome size; the coding sequences (CDS) on the positive (+) and negative (−) strands, along with their functional classification based on Clusters of Orthologous Groups (COGs); the rRNA and tRNA genes on both strands; the GC content; and the GC skew. Arrows indicate the location of prophages and ribosomally synthesized and post-translationally modified peptides (RiPPs) within the genome. (B, C) These panels show the biosynthetic gene clusters (BGCs) identified within the genome using antiSMASH (v7.1), where **(B)** represents the BGCs on the chromosome and **(C)** on the plasmid.

Its genome comprises 2,741,738 base pairs (bp) with a GC content of 38.1%, encoding 2,632 coding sequences (CDSs), 91 tRNAs, 4 non-coding RNAs (ncRNAs), and 108 pseudogenes (Supplementary Tables SM1, SM2). The complete genome sequence has been deposited in GenBank under accession numbers CP162859 and CP162860. The main chromosome and plasmid (Figure 5A) of LBM-M3D harbors a RiPP-like regions and bacteriocin-like sequences corresponding to enterolysin A, mundticin ATO6, enterocin SE-K4, and enterocin P, along with two bacteriocin immunity proteins. These regions also include auxiliary genes associated with regulation, transport, and post-translational processing (Figures 5B,C).

To assess the conservation of the identified bacteriocin sequences across the *E. lactis* species, we performed comparative sequence analyses using BLASTp and MMseqs2 (v.18-8cc5c) against a dataset comprising 100 complete *E. lactis* genomes (Dos Anjos Almeida et al., 2025).

Enterocin P exhibited the highest conservation, with 47 genomes showing 100% amino acid identity and an average identity of 94.5% across the dataset. Enterocin SE-K4 showed a more heterogeneous conservation pattern, with 65 genomes displaying 100% identity but an average identity of 70% across the dataset. Mundticin ATO6 showed lower conservation, with an average identity of 68% and only three genomes exhibiting 100% identity, and 92.98% identity with AAR26473.1 (*E. mundtii*), suggesting that this bacteriocin may be less prevalent or more variable within the species. Enterolysin A demonstrated high intraspecies conservation (average 94%), but only 40.8% identity to the canonical enterolysin A from *E. faecalis*, indicating sequence divergence relative to the canonical reference while retaining conserved peptidoglycan hydrolase domains.

The main chromosome of LBM-M3D encodes multiple multidrug efflux transporters, including three *vanZ* gene copies associated with low-level glycopeptide resistance (e.g., vancomycin, teicoplanin), and *efrA*, *efrB*, and *efmA* genes associated with resistance to erythromycin (macrolides), ciprofloxacin (fluoroquinolones), tetracycline, and chloramphenicol. Additional resistance genes include *aac6’-PRIME* (encoding an aminoglycoside N-acetyltransferase) and *msrC* (mediating erythromycin resistance). *TetR/AcrR* transcriptional regulators, which control the detoxification and efflux systems, were also identified. These regulators repress target operons under basal conditions and activate resistance/detoxification pathways upon inducer binding (e.g., antibiotics, toxins, or stressors like detergents, heavy metals, or plant-derived compounds). Virulence-associated genes detected in the chromosome include three *hemolysin*-related genes, the cell wall-anchored protein SgrA, and a collagen adhesin precursor Acm (Table 3). Although the *acm* gene shared 86% coverage and 96% identity with the database entry for virulence factors (VFDB ID: AAN12397), it was structurally annotated as a pseudogene in LBM-M3D. The genomic context of these antibiotic resistance and virulence-associated determinants was mapped, showing their location in stable chromosomal regions without association with flanking mobile genetic elements, such as transposons, integrons, or insertion sequences [EFSA Panel on Additives and Products or Substances used in Animal Feed (FEEDAP) et al., 2018]. Two intact tailed prophages were also identified. No plasmid-associated antibiotic resistance or virulence genes were detected (Supplementary Table SM3).

## 4 Discussion

The excessive use of antibiotics as growth promoters in the agricultural industry has led to significant challenges, particularly the emergence of antibiotic-resistant bacteria that pose serious public health risks (Butaye et al., 2003; Ma et al., 2021; Bermúdez-Puga et al., 2025). These resistant strains compromise animal health and welfare while reducing productivity. In the swine sector, probiotic bacteria offer one of the most promising strategies to mitigate these issues, providing a viable alternative to decrease antibiotic use and its negative impacts (Leistikow et al., 2022; Yang et al., 2024). Accordingly, the primary objective of this study was to isolate a LAB with probiotic potential from the swine gut microbiota, thereby increasing the likelihood of host-associated compatibility with the indigenous microbiota when administered as a supplement. Furthermore, we sought a strain capable of producing bacteriocins and/or other antimicrobial peptides with efficacy against pathogens relevant to swine production, which could confer additional functional value compared with conventional probiotic candidates.

The escalating challenge of antimicrobial resistance (AMR) in swine production necessitates sustainable alternatives to antibiotics. In this study, LBM-M3D was isolated from swine cecal samples and identified through whole-genome sequencing, achieving an average nucleotide identity (ANI) of >97.8% with validated *E. lactis* strains. This taxonomic precision is critical, as *E. lactis* is genetically distinct from *E. faecium* (ANI < 95.5%), a species excluded from current Qualified Presumption of Safety (QPS) status due to safety concerns in certain strains [EFSA Panel on Biological Hazards (BIOHAZ) et al., 2025]. This is attributed to its ambiguous taxonomic classification and the presence of potentially deleterious traits in certain strains within the *E. faecium* taxonomic unit (TU).

Although *E. lactis* is not currently included in the EFSA QPS list due to insufficient species-level safety information, recent comparative genomic studies suggest that this species may carry a lower burden of acquired antimicrobial resistance, virulence-associated determinants, and mobile genetic elements than closely related enterococci [EFSA Panel on Biological Hazards (BIOHAZ) et al., 2023; Choi et al., 2024]. Therefore, new isolates of *E. lactis* still require careful strain-level safety assessment. In this context, the present study investigated key safety-related features of LBM-M3D using complementary *in vitro* and *in silico* analyses, contributing strain-level data relevant to the future regulatory evaluation of this species.

The strain’s safety profile was assessed through phenotypic and genomic analyses. Phenotypically, the strain was susceptible to eight out of nine clinically important antibiotics tested by broth microdilution, while LBM-M3D cells demonstrated growth at erythromycin concentrations above the cutoff established by EFSA guidelines for enterococci and were therefore classified as resistant to this antibiotic. In accordance with EFSA BIOHAZ Panel recommendations [EFSA Panel on Biological Hazards (BIOHAZ) et al., 2025], whole-genome sequencing (WGS) analysis of LBM-M3D revealed the presence of the erythromycin resistance-associated gene *efmA* (100% coverage and identity with AB467372.1 in the CARD database). This gene encodes a chromosomally located Major Facilitator Superfamily (MFS) transporter permease, a transmembrane protein associated with macrolide and fluoroquinolone resistance in *Enterococcus* spp. (Wan et al., 2021). Although *efmA* is typically characterized in *E. faecium*, its occurrence in LBM-M3D was consistent with a chromosomally encoded, intrinsic-like determinant rather than a plasmid-associated acquired resistance gene.

The safety characterization of LBM-M3D provides important strain-level data that contributes to the scientific understanding and regulatory positioning of this species, especially since EFSA recently noted insufficient species-level safety information for *E. lactis* in its QPS evaluations [EFSA Panel on Biological Hazards (BIOHAZ) et al., 2025]. In this context, our findings regarding *efmA*, *aac*(6′)-PRIME, and *vanZ* are consistent with recent comparative genomics analyses that identified these genes as conserved components of the *E. lactis* core genome (Ahmed et al., 2023; Lu et al., 2023). Pangenomic data focused specifically on this species reveal that the identified resistance determinants, such as *aac*(6′) and *msr*(C), are highly conserved components of the core genome, being present in 100 and 96%, respectively, of publicly available *E. lactis* genomes (Dos Anjos Almeida et al., 2025). Analysis of their genetic environment indicated chromosomal localization without obvious association with flanking mobile genetic elements, such as transposons, integrons, or insertion sequences (IS), which suggests a reduced risk of horizontal transfer compared with plasmid- or transposon-associated AMR determinants. Furthermore, bioinformatic analysis of several *E. lactis* strains revealed a set of 160 genes potentially associated with antimicrobial resistance (AMR), including determinants associated with chloramphenicol, vancomycin, clindamycin, erythromycin, quinupristin–dalfopristin, and rifampicin resistance (Lu et al., 2023).

Consequently, susceptibility to a specific group of antibiotics is an essential component of safety assessment for bacteria intended for probiotic use. The absence of transferable antibiotic resistance genes, particularly those associated with plasmids and conjugative transposons, is a critical factor according to regulatory agencies (Charteris et al., 1998; Sharma et al., 2017). Currently, acquired AMR genes are understood as resistance determinants introduced through horizontal gene transfer and potentially located either on mobile elements or integrated into the chromosome. In contrast, intrinsic AMR genes are inherent to a bacterial species and shared by the vast majority of its wild-type strains, generally posing a lower safety risk. Thus, distinguishing intrinsic-like chromosomal determinants from acquired and potentially mobile resistance genes is essential for interpreting the safety profile of candidate probiotic strains.

Virulence-associated genes, such as the *sgrA* gene, which encodes a surface protein involved in binding extracellular matrix proteins and biofilm formation (Hendrickx et al., 2009), as well as the *acm* gene, which participates in collagen adhesion (Nallapareddy et al., 2008), were identified. Although these virulence factors are typically identified in pathogenic strains to promote their colonization and propagation, nevertheless, these genes are intrinsic in *Enterococcus* species and were described as low-pathogenicity indicators. Recent comparative genomic characterizations of the *E. lactis* species support that these sequences are part of the core genetic repertoire, functioning primarily as commensal colonization factors rather than active virulence determinants (Ahmed et al., 2023; Lu et al., 2023). While EFSA guidelines provide a framework, they do not explicitly classify virulence genes as intrinsic. Thus, our identification of chromosomal *sgrA* and *acm* characterizes LBM-M3D as a low-pathogenicity isolate. Indeed, the *in vitro* properties of these factors are consistent with the strain’s ability to achieve transitory colonization of the gastrointestinal tract, promoting pathogen competition. However, *in vivo* safety and efficacy studies as required by the EFSA FEEDAP remain an essential requirement to validate these genomic predictions before making functional claims [Rolfe, 2000; EFSA Panel on Additives and Products or Substances used in Animal Feed (FEEDAP) et al., 2018; Fu et al., 2022].

Genome sequencing of *E. lactis* LBM-M3D revealed the presence of three genes associated with hemolysin production, which are intrinsic to this bacterial species. The identified Hemolysin III family genes have been reported in both pathogenic and non-pathogenic bacteria in the literature (Baida and Kuzmin, 1996; Vallejo et al., 2020). The classification of these hemolysin-related genes as intrinsic determinants is corroborated by safety evaluations of the entire species, which found no correlation between their genomic presence and a hemolytic phenotype in the majority of *E. lactis* isolates (Ahmed et al., 2023). The LBM-M3D strain demonstrated γ-hemolytic activity in blood agar assays, indicating the absence of a hemolytic phenotype. This finding could be explained by the partial absence of genes within the operon responsible for hemolysin biosynthesis or by mutations in these genes, resulting in an inactive or incomplete form of hemolysin that prevents the expression of hemolytic activity (Semedo et al., 2003; Vallejo et al., 2020; Ahmed et al., 2023). The presence of hemolysin-related genes in the absence of a hemolytic phenotype is consistent with observations from other *E. lactis* strains and may be explained by partial gene deletions or mutations within the hemolysin operon (Semedo et al., 2003; Ahmed et al., 2023). This underscores the importance of combining genomic prediction with phenotypic validation when assessing safety, as *in silico* identification of virulence-associated sequences does not necessarily translate to functional pathogenicity. While the presence of *sgrA* and hemolysin genes warrants caution, their common occurrence in commensal *E. lactis* genomes and the lack of phenotypic expression in LBM-M3D suggest they are unlikely to contribute to pathogenic potential under the conditions evaluated.

Despite the robust chromosomal assembly achieved via PacBio HiFi sequencing, a potential limitation is the incomplete recovery of the mobilome due to the size selection step during library preparation, which can result in the loss of small plasmids (<5 kb) (Johnson et al., 2023). Complementary strategies, such as short-read sequencing, were not employed to capture small extrachromosomal elements. However, our approach successfully resolved the 160 kb plasmid I, which harbored neither virulence factors nor acquired antimicrobial resistance. In addition, the stable chromosomal localization of the identified resistance determinants minimizes the safety risks associated with potentially undetected small replicons. However, fully characterizing the LBM-M3D mobilome will require future studies utilizing complementary sequencing approaches.

LBM-M3D’s resilience to gastrointestinal stressors (pH 3.0–8.0, 0.1–0.5% bile salts) and bacteriocin-mediated pathogen suppression support its further evaluation as a multifunctional probiotic candidate. These findings suggest that LBM-M3D exhibits relevant *in vitro* adaptability, comparable to that observed in other *Enterococcus* species (Bhardwaj et al., 2010; Nami et al., 2014). Its pH tolerance is compatible with survival under acidic stress, while bile salt resistance suggests potential persistence under intestinal-like conditions (Corcoran et al., 2005). The strain’s ability to inhibit both endemic swine pathogens (*S. suis*, *S. hyicus*) and zoonotic risks (*L. monocytogenes*) aligns with One Health objectives, bridging animal health and food safety.

Beyond its antimicrobial properties, recent genomic characterization of LBM-M3D has revealed additional functional traits that may contribute to its probiotic potential, including genes associated with pectin degradation and the biosynthesis of B-complex vitamins (riboflavin, folate) and menaquinone (vitamin K2) (Dos Anjos Almeida et al., 2025). This metabolic versatility, combined with its bacteriocinogenic activity, supports the potential of LBM-M3D for future probiotic development, suggesting that it may contribute to pathogen suppression and modulation of the intestinal environment through the production of bioactive metabolites.

The cell-free supernatant (CFS) of LBM-M3D exhibited proteinaceous or peptide-associated antimicrobial activity, as supported by the reduction or loss of inhibition after protease treatment, against *L. monocytogenes*, *S. suis*, *S. hyicus*, and *E. faecalis*. In contrast, the absence of inhibition against *E. coli* aligns with the typical Gram-positive bacteria specificity of bacteriocins. Notably, growth kinetics revealed that pathogens treated with CFS eventually reached a higher final optical density (OD_600_) compared to the positive controls. This phenomenon may be explained, at least in part, by the additional nutrient supply from the residual MRS medium and intracellular metabolites in the CFS. However, this interpretation should be made cautiously, as OD₆₀₀ measurements do not discriminate between viable and non-viable cells and may overestimate bacterial growth due to cell debris or morphological changes. Therefore, complementary viability-based assays (e.g., CFU enumeration) are required to accurately determine whether the observed effects correspond to true regrowth or delayed recovery. Once the initial inhibitory pressure of the bacteriocins subsides, the pathogens may exploit these remaining substrates for more robust proliferation, a behavior consistent with metabolite provisioning models in closed systems (Pherribo and Taga, 2023; Todorov and Dicks, 2006).

This inhibitory profile is consistent with genomic analysis, which identified three class II bacteriocin-like sequences: enterocin SE-K, enterocin P, and mundticin ATO6 (Cintas et al., 1997; Eguchi et al., 2001; Kawamoto et al., 2002; Teymurazov et al., 2021). These peptides harbor the conserved ‘YGNGV’ motif at their N-terminal domain, essential for electrostatic membrane binding, and a hydrophobic C-terminal domain responsible for pore formation in the target cell membrane (Johnsen et al., 2000; Chen et al., 1997; Axelsson and Holck, 1995). Additionally, the class III bacteriocin enterolysin A was identified in the chromosome. Enterolysin A is a large protein with peptidoglycan hydrolase activity that leads to cell lysis (Khan et al., 2013). Together, these predicted antimicrobial determinants suggest that LBM-M3D carries a diverse bacteriocinogenic repertoire that may contribute to the inhibition of Gram-positive pathogens. The coexistence of multiple bacteriocin systems may provide functional redundancy or synergistic effects, potentially enhancing antimicrobial robustness under different environmental conditions.

The simultaneous presence of multiple bacteriocin-like determinants in LBM-M3D may help explain the diverse antimicrobial effects observed across different swine pathogens. This repertoire is consistent with pathogen-specific growth responses, ranging from complete suppression of detectable *S. suis* growth by OD_600_ over 24 h to delayed growth of *L. monocytogenes*, which exhibited a 5-h lag relative to the positive control. However, OD600-based growth kinetics alone do not allow a definitive distinction between bacteriostatic and bactericidal effects, and viable-cell counting will be required to confirm whether the activity against *S. suis* is bactericidal. Although enterolysin A is known for lytic activity against Gram-positive bacteria, its expected heat-lability and large molecular size make it unlikely to be the sole driver of the heat-resistant inhibitory activity observed in the crude CFS. Therefore, the strong inhibition of *S. suis* may result from one or more thermostable antimicrobial components, including class II bacteriocins such as mundticin ATO6, enterocin SE-K4, and enterocin P, or other yet-uncharacterized stable molecules present in the CFS. In contrast, the delayed growth observed for *L. monocytogenes* and *E. faecalis* is compatible with membrane-targeting bacteriocin-like activity, although this mechanism remains to be experimentally confirmed. The more limited impact on *S. hyicus* may reflect species-specific tolerance mechanisms, such as differences in membrane composition or efflux activity. This broad Gram-positive inhibitory activity is consistent with prior studies reporting antagonistic effects of enterococcal bacteriocins against *Enterococcus* spp., *Clostridium* spp., *L. monocytogenes*, *Staphylococcus aureus*, *Bacillus subtilis*, and diverse lactic acid bacteria (Cintas et al., 1997; Bennik et al., 1998; Eguchi et al., 2001; Shori, 2017). Although the genome encodes several class II bacteriocins, including mundticin ATO6, enterocin SE-K4, and enterocin P, the identity of the active antimicrobial molecule detected in the purified fraction was not established in the present study. Therefore, whether any of these predicted bacteriocins is responsible for the observed antimicrobial activity remains to be experimentally confirmed.

It is worth noting that the structural organization and identity of the bacteriocin biosynthetic gene clusters (BGCs) described herein are consistent with the broader enterocin HF-like and class II bacteriocin clusters previously mapped in the *in-silico* framework by Dos Anjos Almeida et al. (2025) for the same *E. lactis* strain. Specifically, the sequence annotated as enterocin SE-K4 (76.1% identity to the BAGEL4 reference) corresponds to an enterocin HF-like peptide, consistent with the independent genomic characterization that reported 98% identity to enterocin HF (UniProt: P86183).

Future applications of LBM-M3D could include direct oral administration or bacteriocin encapsulation to enhance stability and targeted delivery. For instance, microencapsulation in alginate matrices has proven effective for similar LAB strains, protecting viability during feed processing and gastric passage (Shori, 2017; Vivek et al., 2023; Frota et al., 2024). Scaling bacteriocin production via fermentation, coupled with cost-effective purification methods, will be critical for industrial adoption (Lajis, 2020). According to the consensus statement of the International Scientific Association for Probiotics and Prebiotics, the definition and assessment of probiotics require rigorous criteria (Hill et al., 2014). In this context, our findings provide preliminary evidence that will guide future studies to further validate the probiotic potential of LBM-M3D. However, *in vivo* trials are essential to validate gut colonization efficiency, pathogen suppression in live models, and long-term safety. Comparative studies against established probiotics (e.g., *Bacillus subtilis* or *Pediococcus* spp.) could further delineate its competitive advantages in swine microbiota modulation.

## 5 Conclusion

The LBM-M3D strain, isolated from the swine gastrointestinal tract, displays significant probiotic-associated characteristics, making it a promising candidate for further development and evaluation as a feed-associated probiotic in swine production. Its bacteriocinogenic potential and antimicrobial activity against pathogens relevant to swine health support its possible application as part of sustainable strategies aimed at reducing reliance on antibiotics as growth promoters, thereby contributing to efforts to mitigate antimicrobial resistance (AMR). Additionally, the detection of a low-molecular-weight candidate antimicrobial molecule in the active anti-*Listeria* fraction suggests the presence of a previously uncharacterized inhibitory compound, expanding the potential for future biotechnological applications.

A promising avenue for future exploration is the use of semi-purified or encapsulated antimicrobial fractions/bacteriocin-like compounds LBM-M3D as nutraceuticals or therapeutic agents to enhance health and prevent infections. Upcoming studies should aim to structurally characterize this candidate molecule, including by LC–MS/MS sequencing, evaluate its biological efficacy in relevant models, and rigorously assess its safety and encapsulation strategies. Furthermore, future application of LBM-M3D or its antimicrobial compounds in swine production may represent a practical and impactful strategy, promoting healthier livestock, reducing pathogenic bacteria, and supporting sustainable practices in the swine industry.

## Data Availability

The datasets generated and/or analyzed during the current study are available in the NCBI GenBank under accession numbers CP162859 and CP162860.

## Footnotes

1
https://github.com/moshi4/ANIclustermap

2
https://github.com/tseemann/ABRICATE

3
https://inkscape.org
