# Supplementary material for: Genomic safety, bacteriocinogenic potential, and probiotic-associated traits of Enterococcus lactis LBM-M3D isolated from the swine gastrointestinal tract
Source: Front Microbiol. 2026 Sep 3;17:1920982. doi: 10.3389/fmicb.2026.1920982 (PMC13582303; doi:10.3389/fmicb.2026.1920982)
Supplement: Supplementary file 1 [file Table_1.DOCX]

**Supporting material**

Supplementary Table **SM1**. Reference genomes used for Average Nucleotide Identity (ANI) analysis.

| Species | *Enterococcus lactis* | *Enterococcus faecium* |
| --- | --- | --- |
| Access Number | GCA_015767715 | GCA_009734005 |
| Access Number | GCA_019203145 | GCA_029023785 |
| Access Number | GCA_019343125 | GCA_001720945 |
| Access Number | GCA_019880345 | GCA_009697285 |
| Access Number | GCA_019967715 | GCA_019977575 |
| Access Number | GCA_020268645 | GCA_900639535 |
| Access Number | GCA_023612275 | GCA_002007625 |
| Access Number | GCA_030490365 | GCA_900639715 |
| Access Number | GCA_033954875 | GCA_900639485 |
| Access Number | GCA_033954895 | GCA_002025045 |
| Access Number | GCA_033954915 | GCA_019977495 |
| Access Number | GCA_036324345 | GCA_023204955 |
| Access Number | GCA_036669985 | GCA_016415405 |
| Access Number | GCA_040745045 | GCA_038441135 |
