# Supplementary material for: Genomic safety, bacteriocinogenic potential, and probiotic-associated traits of Enterococcus lactis LBM-M3D isolated from the swine gastrointestinal tract
Source: Front Microbiol. 2026 Sep 3;17:1920982. doi: 10.3389/fmicb.2026.1920982 (PMC13582303; doi:10.3389/fmicb.2026.1920982)
Supplement: Supplementary file 2 [file Table_2.DOCX]

**Supporting material**

Supplementary Table **SM2.** Genomic features and assembly metrics of Enterococcus lactis LBM-M3D.

| General information | Value |
| --- | --- |
| Organism | *Enterococcus lactis* |
| Strain | LBM-M3D (LBMFMM3D) |
| Bioproject | PRJNA1121381 |
| Biosample | SAMN41748423 |
| Genbank accession (chromosome) | CP162859 |
| Genbank accession (plasmid) | CP162860 |
| Assembly quality metrics |  |
| Sequencing platform | PacBio HiFi REVIO |
| Assembly depth | 109x |
| N50 | 2,450,530 bp |
| L50 | 2 |
| Checkm completeness | 99.42% |
| Checkm contamination | 0.27% |
| Busco completeness | 99.2% |
| Genomic features (annotation via pgap) |  |
| Total genome size | 2,741,108 bp |
| Chromosome size | 2,581,108 bp |
| Plasmid i size | 160,000 bp (160 kb) |
| Global gc content | 38% |
| Total genes | 2,54 |
| Protein-coding sequences (cds) | 2,45 |
| Rrna genes | 18 (6 5S, 6 16S, and 6 23S) |
| Trna genes | 69 |
| Ncrna genes | 4 |
| Pseudogenes | 80 |
| Intact prophage regions | 2  (Enterococcus_phage_vB_EfaS_IME197, Enterococcus_phage_EFC-1) |
