# Supplementary material for: Genomic safety, bacteriocinogenic potential, and probiotic-associated traits of Enterococcus lactis LBM-M3D isolated from the swine gastrointestinal tract
Source: Front Microbiol. 2026 Sep 3;17:1920982. doi: 10.3389/fmicb.2026.1920982 (PMC13582303; doi:10.3389/fmicb.2026.1920982)
Supplement: Supplementary file 3 [file Table_3.DOCX]

**Supporting material**

**Table SM**3**.** Genomic characteristics of *Enterococcus lactis* strain LBM-M3D sequenced.

| **Features** | ***E lactis* LBM-M3D** |
| --- | --- |
| Genomic size (bp) | 2,741,738 |
| - main chromosome (bp) | 2,581,108 |
| Sequencing Depth (mean) | 109x |
| GC content (%) | 38.1 |
| Genes (total) | 2.632 |
| CDSs (total) | 2.524 |
| tRNA | 91 |
| 5S rRNA | 6 |
| 16S rRNA | 6 |
| 23S rRNA | 6 |
| ncRNA | 4 |
| Pseudogenes | 108 |
| tRNA | 91 |
| 5S rRNA | 6 |
| Prophage regions (intacts) | 2 |
| - prophage 1 (kb) | 41 |
| - prophage 2 (kb) | 43.6 |
| Plasmids | 1 |
| - plasmid 1 (bp) | 160,630 |
|  | **chr encoded** |
| Antibiotic Resistance-related genes | 3x *vanZ* (glycopeptide antibiotic) |
|  | 5x multidrug efflux transporters |
|  | 12x TetR/AcrR family transcriptional regulator |
|  | tetracycline resistance MFS efflux pump |
|  | *AAC6-PRIME* ,aminoglycoside N-acetyltransferase AAC(6') |
|  | *efmA* multidrug efflux MFS transporter EfmA |
|  | ABC-F type ribosomal protection protein Msr |
|  | dltABCD operon: Cationic antimicrobial peptide (CAMP) resistance |
|  | **chr encoded** |
| Bacteriocins genes | Enterolysin A |
|  | Mundticin ATO6 |
|  | peptide cleavage/export ABC transporter |
|  | bacteriocin immunity protein |
|  | enterocin immunity protein |
|  | **plasmid encoded** |
|  | Enterocin SE-K4 |
|  | Enterocin P |
|  | class II bacteriocin |
|  | bacteriocin immunity protein |
|  | **chr encoded** |
| Virulence-related genes | hemolysin III family protein |
|  | 2x hemolysin family protein |
|  | cell wall anchored protein SgrA |
|  | collagen adhesin precursor Acm |
| CheckM (completeness/contamination) | 99.42 / 0.50 |
| BUSCOs (completeness) | 99.2 |
| ANI | 97.88 |
